# Pain Self-Management for Veterans: Development and Pilot Test of a Stage-Based Mobile-Optimized Intervention

**DOI:** 10.2196/medinform.7117

**Published:** 2017-10-17

**Authors:** Sara S Johnson, Deborah A Levesque, Lynne E Broderick, Dustin G Bailey, Robert D Kerns

**Affiliations:** ^1^ Pro-Change Behavior Systems, Inc South Kingstown, RI United States; ^2^ VA Connecticut Healthcare System, Yale University Research Psychologist, Pain Research, Informatics, Multimorbidities and Education (PRIME) Center Professor of Psychiatry, Neurology and Psychology West Haven, CT United States

**Keywords:** pain management, self-management, mobile health, mhealth

## Abstract

**Background:**

Chronic pain is a significant public health burden affecting more Americans than cardiovascular disease, diabetes, and cancer combined. Veterans are disproportionately affected by chronic pain. Among previously deployed soldiers and veterans, the prevalence of chronic pain is estimated between 44% and 60%.

**Objective:**

The objective of this research was to develop and pilot-test *Health eRide: Your Journey to Managing Pain*, a mobile pain self-management program for chronic musculoskeletal pain for veterans. Based on the transtheoretical model of behavior change, the intervention is tailored to veterans’ stage of change for adopting healthy strategies for pain self-management and their preferred strategies. It also addresses stress management and healthy sleep, two components of promising integrated treatments for veterans with pain and co-occurring conditions, including posttraumatic stress disorder (PTSD) and traumatic brain injury. In addition, Health eRide leverages gaming principles, text messaging (short message service, SMS), and social networking to increase engagement and retention.

**Methods:**

Pilot test participants were 69 veterans recruited in-person and by mail at a Veterans Health Administration facility, by community outreach, and by a Web-based survey company. Participants completed a mobile-delivered baseline assessment and Health eRide intervention session. During the next 30 days, they had access to a Personal Activity Center with additional stage-matched activities and information and had the option of receiving tailored text messages. Pre-post assessments, administered at baseline and the 30-day follow-up, included measures of pain, pain impact, use of pain self-management strategies, PTSD, and percentage in the Action or Maintenance stage for adopting pain self-management, managing stress, and practicing healthy sleep habits. Global impressions of change and program acceptability and usability were also assessed at follow-up.

**Results:**

Among the 44 veterans who completed the 30-day post assessment, there were statistically significant pre-post reductions in pain (*P*<.001) and pain impact (*P*<.001); there was some reduction in symptoms of PTSD (*P*=.05). There were significant pre-post increases in the percentage of participants in the Action or Maintenance stage for adopting pain self-management (*P*=.01) and for managing stress (*P*<.001) but not for practicing healthy sleep habits (*P*=.11). The global impressions of change measure showed that a majority had experienced some level of improvement. User ratings of acceptability were quite high; ratings of usability fell slightly below the mean for digital programs.

**Conclusions:**

Preliminary data demonstrate the potential impact of the Health eRide program for chronic musculoskeletal pain for veterans. The results underscore that simultaneously addressing other behaviors may be a promising approach to managing pain and comorbid conditions. Additional formative research is required to complete development of the Health eRide program and to address areas of usability requiring improvement. A randomized trial with longer follow-up is needed to demonstrate the program’s long-term effects on pain and pain self-management.

## Introduction

### Pain and Pain Self-Management

Chronic pain is a significant public health burden affecting more Americans than cardiovascular disease, diabetes, and cancer combined [1]. The economic toll of chronic pain is approximately US $635 billion annually. Veterans are disproportionately affected by chronic pain [2,3]. The prevalence of chronic pain among previously deployed soldiers and veterans is estimated between 44% and 60% [4,5], compared with 26% in a primary care sample [6]. Among veterans, pain is the most costly of all disorders treated in the Veterans Health Administration (VHA) facilities [7]. Chronic pain is particularly common among the veterans of Operations Iraqi Freedom (OIF), Enduring Freedom (OEF), and New Dawn. Furthermore, the co-occurrence of pain and posttraumatic stress disorder (PTSD), traumatic brain injury (TBI), and all three conditions (postdeployment multisymptom disorder, or PMD) is well documented [3,8,9] and can complicate and reduce the effectiveness of treatment of pain [10-12]. The proponents of integrated treatment for PMD or the co-occurrence of pain with either PTSD or TBI are advocating for innovative delivery of interventions that can address multiple conditions [8].

The ongoing personal, social, and economic burden of pain indicates that existing treatment approaches are insufficient. In addition, there is growing concern about the reliance on chronic opioid therapy for chronic pain [13], with mounting data questioning its efficacy and safety [14-18], particularly for veterans [19]. The 2011 Institute of Medicine Blueprint for Relieving Pain in America calls for a population-level pain management strategy; the promotion of self-management; reducing disparities among vulnerable subgroups; and the tailoring of pain care for each patient [1]. The need to increase the quality, variety, and accessibility of nondrug, evidence-based pain self-management skills is even more urgent for veterans, given that they are also disproportionately affected by the current opioid crisis in the United States [2,5].

There are numerous barriers to pain treatment for veterans—such as limited availability of therapists adequately trained in pain self-management [20], cost [20-23], and the distance or logistics of traveling to appointments [20,21]. Pain treatment is further hindered by limited or inadequate individual tailoring of treatment and an overreliance on ineffective and potentially risky treatments, including the use of opioid analgesics and surgical procedures [24]. Thus, veterans with chronic pain are at risk for a lifetime of increasingly progressive disability. The costs of that disability and its treatment could approach US $5 trillion [3].

Reviews have consistently demonstrated the effectiveness of exercise [25-27] and cognitive behavioral therapy (CBT) [28,29] for the treatment of pain. CBT encourages the use of cognitive (eg, coping self-statements) and behavioral (eg, activity pacing) pain coping skills. Interventions that increase reliance on those skills and adopt a biopsychosocial approach that acknowledges that biological, psychological, and social factors influence how pain is experienced and managed can significantly reduce pain, disability, and depressive symptoms [30].

Mobile technologies offer a promising approach to delivering pain self-management treatments incorporating CBT principles. Mobile-delivered interventions can reduce barriers related to access to treatment; they are convenient, enable a high degree of individual tailoring, and can be delivered with fidelity. At least 89% of adults in the United States have access to the Internet [31], and 79% own a smartphone [32]. Furthermore, among groups with historically less Internet access, the digital divide is shrinking. Whereas 44.0% of a sample of 266 veterans aged 65 years and older reported not having access to the Internet at home, nearly 50% had at least one close social tie whom they could ask to use a device, and 70% had at least one social tie whom they would ask for help accessing the Internet [33].

Research assessing the efficacy of mobile or Web-based pain self-management interventions or apps show, on average, positive preliminary results for pain severity, coping self-statements, and other outcomes [34,35]. However, a major problem with existing interventions is that they tend to neglect individual differences in motivation and readiness to adopt self-management strategies [36], have limited input from end users in the development calling into question their usability [37], fail to address other comorbid conditions [38], and are not based on evidence-based practices [39]. Another limitation is that no veteran-specific intervention could be identified.

Although mobile apps that promote self-management have the potential to speed the adoption of individualized, evidence-based, biopsychosocial treatments for pain [40], those developed to date have largely failed to deliver on that promise. A review of 195 mobile phone apps for pain management found serious limitations in those currently available: only 3% incorporated any evidence-based guidelines or principles from CBT [39]. None have been tested in rigorous clinical trials [39,40], and none developed specifically for veterans could be identified.

The primary objective of this research was to develop and conduct a pilot test of a theoretically grounded, mobile- optimized, Internet-based, interactive pain self-management program for veterans with chronic musculoskeletal pain. The program titled *Health eRide: Your Journey to Managing Pain* was designed to address the limitations of existing apps for pain self-management. The Health eRide intervention, developed specifically for veterans (1) relies on a participatory approach to design, eliciting veterans’ input and feedback at each stage of the intervention’s development; (2) integrates evidence-based practices for pain self-management; (3) is tailored to end users’ readiness to adopt those best practices; and (4) helps to address two comorbid conditions—PTSD and TBI—by including health behavior change messages that promote two core elements of promising integrated treatment for PMD: stress management and adoption of healthy sleep practices. In addition, the intervention leverages SMS text messaging (short messaging service, SMS), social networking, and gaming principles to increase engagement and retention. The pilot study reported here was conducted as a preliminary test of the program’s potential impact on pain and other key outcomes among veterans experiencing pain.

### Health eRide Intervention

#### Intervention Development

Intervention development was guided by the VHA’s National Pain Management Strategy’s recommendation to focus on innovative patient education programs, deliver cost-effective pain care, increase satisfaction with pain care, and ensure that veterans’ needs are addressed [41]. It was also decided at the outset that the intervention would be tailored to veterans’ readiness to self-manage pain, as well as their preferences regarding specific pain self-management strategies. The transtheoretical model (TTM) provided the theoretical framework. The TTM explains how individuals progress through a series of five stages of change: precontemplation (not intending to take action); contemplation (intending to take action in the next 6 months); preparation (intending to take action in the next 30 days); action (made the behavior change less than 6 months ago); or maintenance (made the behavior change more than 6 months ago) [42]. The other constructs by TTM—decisional balance, self-efficacy, and processes of change—are systematically related to stages in predictable ways [43-45]. The relationship between stage and these behavior change constructs provide an evidence-based framework for developing and delivering tailored feedback that is more likely to be remembered [46,47]; to be discussed with others [48]; to be considered personally relevant, interesting, and credible [48-50]; and to change behavior [48-50]. TTM-based interventions have been found effective across dozens of behaviors and populations [49,51,52].

Using a participatory design process, formative research elicited input from a panel of veteran advisors, experts, and end users to ensure that the program was perceived as meaningful, understandable, and useful; that its flow was easy to navigate and engaging; that the look and feel were attractive; and that the content was tailored to the veteran culture. Furthermore, input was sought on the most effective manner in which to integrate social networking, principles of gamification, and SMS text messaging. Throughout the development process, end-user interviews, focus groups, and usability testing were conducted to ensure that the program was accessible and acceptable. The content was written in plain language at a 7th grade reading level or less, and all content was reviewed with Health Literacy Advisor software distributed by Health Literacy Innovations, LLC.

#### Principles of Gamification

Efforts were made to maximize engagement and satisfaction with Health eRide by incorporating principles of gamification. The literature [53,54] and gamification experts stressed that gamification tactics must activate meaning, mastery, and autonomy to be effective. To increase meaning and personal relevance, the opening screens of the program ask users to identify their most important reason for managing pain. The options in the list (eg, get back to activities I love, feel more in control) had been generated by interview and focus group participants and veteran advisors. Users also have the option of uploading an image of their reason (eg, a picture of their children). Users are also asked to select an avatar to represent them throughout the program. They can select an avatar from a list provided or upload an image of their own.

Mastery, which is derived from a sense of progressing to a goal or achieving something, was promoted in several ways. The Health eRide Personal Activity Center (PAC), described below, is structured as a subway map that the user must navigate to reach their final destination (ie, their main reason for managing their pain). Once an activity is completed, additional “stations” (ie, activities) become available, enabling the user to proceed closer to their final destination. The avatar moves down the subway map, and the user’s progress is reflected by the accumulation of tickets in a ticket kiosk.

#### Program Flow

**Figure 1:**
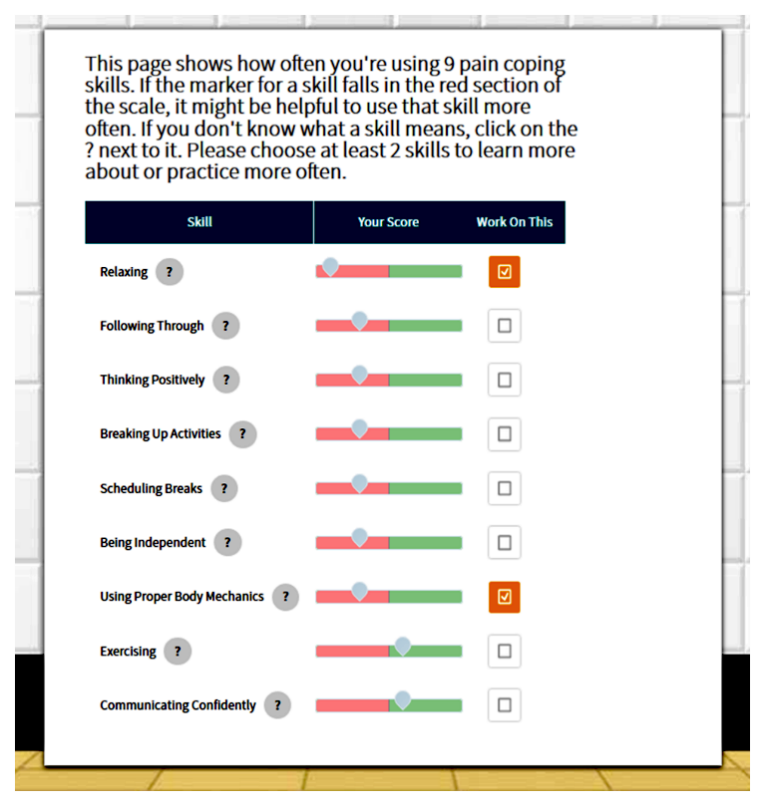
Sample pain coping skills feedback from the Health eRide program.

After inviting program users to select a primary reason for managing pain and to select an avatar, the Health eRide program delivers assessments of pain and stage of change for pain self-management, along with the Multidimensional Pain Readiness to Change Questionnaire (MPRCQ2) [55-57], which assesses readiness to use each of the nine strategies for pain self-management. Users receive feedback on their stage of change and a “report card” showing how often they use each of the coping strategies assessed in the MPRCQ2 (see Figure 1). They are asked to select at least two pain self-management strategies they would like to learn more about or practice more often. The program then administers TTM measures of decisional balance and self-efficacy for pain self-management as well as stage-matched guidance designed to facilitate progress to the next stage for using healthy strategies for pain self-management or to prevent relapse to an earlier stage.

In the second half of the session, participants receive brief assessments and stage-matched guidance targeting stress management and healthy sleep habits. Given the frequent co-occurrence of chronic pain and other conditions, especially PTSD [58] and TBI [59], additional assessments are administered to detect possible symptoms of these conditions. Participants screening positive for PTSD or TBI receive information on local and national resources.

#### Personal Activity Center

Once users complete the computer tailored intervention (CTI) session, they are brought to their PAC, also known as the Health eRide subway station. The subway station is a collection of 56 interactive activities designed to activate the processes of change that are most appropriate for the user based on his or her stage of change for each behavior. Users began with a “tour” of the station to highlight its features, including the participant’s final destination—his or her most important reason for managing pain, identified at the beginning of the CTI session. The tour is designed to acclimate users to the program’s principles of gamification, including unlocking new stations by completing activities and collecting “tickets” as they make stops at each station. Tickets are also used as an incentive for users to explore different stations, as they can collect additional “punches” on the tickets when they make extra stops (ie, complete additional interactive activities) in the stations.

#### Text Messages

At the beginning of the CTI session, users are presented with the opportunity to opt in to receive tailored text messages for each of the three targeted behaviors. Those opting in receive a text message asking them to “validate” their phone number to initiate the messages. Text message content and delivery schedules are matched to the stage of change for each behavior. Sample text messages include:

As a Veteran, you likely know many people who have or had pain. Think about one of them who could inspire you to manage your pain.

Want to be more alert, make better decisions & fewer mistakes? Get a good night’s sleep. It’s not optional. #zzzs

Stress can make people more prone to pain. If you lower your stress, you can help lower your pain. See PAC activity Get the Facts [short-url].

#### Social Networking

Although full Facebook integration was not feasible for this prototype, users had access to a Health eRide Facebook page, which was regularly updated by the project team with relevant posts and content. Each screen of the Health eRide program, including the subway station PAC, included a link to the Facebook page. In one of the subway stop activities, Share Your Success Story, users are also presented with the opportunity to share their own story on the Facebook page.

### Intervention Pilot Test

The remainder of this report describes a pilot test designed to assess the potential impact of the Health eRide program and its usability and acceptability among a small sample of veterans. In the pilot, eligible participants completed a Health eRide CTI session that included several study measures (eg, measures of pain and stage of change); additional study measures (demographics and military history) were appended to the end of the session. During the next 30 days, participants had access to a PAC with additional stage-matched activities and information and had the option of receiving tailored text messages. Follow-up assessments were administered 30 days following the CTI session.

## Methods

### Recruitment

Pilot test participants were 69 veterans not involved in the formative research. Pilot participants were recruited through in-person and mail recruitment at the Veterans Administration Connecticut Healthcare System (VACHS), community outreach and Facebook, and a Web-based survey company. Eligibility criteria included the following: age of 18 years or older; veteran status; having a chronic musculoskeletal pain rating of 4 or higher on a 0 to 10 numerical scale of pain intensity [60]; having had pain for more than 3 months; and not currently undergoing treatment with a psychologist, psychiatrist, or other mental health professional for a condition such as bipolar disorder, anxiety, or substance abuse.

#### In-Person and Mail Recruitment at the VACHS (n=29)

At the outset, a research assistant worked with pain clinic staff, nurses, and physicians at VACHS to identify potential participants and to promote the study at a community outreach table. In addition, a research assistant recruited potential participants from primary care waiting rooms. In both cases, the research assistant screened for eligibility and eligible participants were provided with a program link, user ID, and temporary password. Participants had the option of completing the baseline assessment and CTI session at the VA, on an iPad (Apple Inc) provided, or at home. were asked to call the VA facility during business hours to complete a phone screening with the research assistant. Eligible participants were provided with the program link and log-in credentials.

#### Community Outreach and Facebook (n=9)

The project team provided flyers to the local Operation Iraqi Freedom (OIF) and Operation Enduring Freedom (OEF) coordinator within the VA, as well as to local university and college veteran representatives; invited Veteran organizations to share recruitment information with the veterans they serve; hung flyers in grocery stores, coffee shops, veterans of foreign wars halls, and other settings; and reached out to personal contacts. Veterans who were interested in participating were asked to call the VA facility during business hours to complete a phone screening with the research assistant. Eligible participants were provided with a program URL and log-in credentials.

Other outreach activities included an 8-day national Facebook ad campaign targeting adults aged between 18 and 65, whose Facebook interests matched keywords, including Iraq and Afghanistan Veterans of America, Wounded Warrior Project, and back pain. Interested Facebook users were linked to an eligibility screener. Although the ad reached 42,811 Facebook users, and 945 of those users clicked through to the eligibility screener, none completed the Web-based eligibility screener.

#### Web-Based Survey Company (n=31)

The final recruitment channel was a Web-based survey company, Survey Sampling International (SSI). Panel members are individuals who agree to receive invitations matched to personal information they provide. SSI sent email invitations to panel members who had reported that they are veterans. Interested members completed a Web-based screener, and those meeting the eligibility criteria were provided with a link and log-in credentials for the Health eRide Program.

#### Participant Demographics and Military History

Participants’ mean age was 50.3 years (SD 12.0); 81% (56/69) were male; 55% (38/69) were white non-Hispanic, 33% (23/69) black non-Hispanic, 9% (6/69) Hispanic, and 3% (2/69) “other”; 62 % (43/69) were married or cohabiting with a partner, 12% (8/69) were single and never married, and 26% (18/69) were separated, divorced, or widowed; 16% (11/69) had no education beyond high school, 35% (24/69) had attended some college, 41% (28/69) had a college degree, and 9% (6/69) had some postgraduate education. Participants had served an average of 8.7 years (SD 7.1) in the military; rank at discharge was enlisted for 50% (34/68) of the participants, senior enlisted for 44% (30/68) and officer for 6% (4/68). About half (48%, 33/69) reported that they had been deployed to Iraq, Afghanistan, the Gulf, Vietnam, and/or Korea, and 22% (15/69) reported that they had been deployed elsewhere.

### Procedure

Participants completed a Health eRide CTI session, which included baseline measures, and were encouraged to complete at least two PAC activities. The PAC remained available for 30 days. For veterans opting to receive text messages, the program also delivered messages for 30 days. Upon completion of their first CTI session, participants received a US $25 gift card or, for SSI participants, US $25 worth of “points” that they could exchange for rewards. Thirty days post baseline, participants were prompted via email to complete a brief follow-up assessment and acceptability survey. Nonrespondents received a reminder call from the VA facility research assistant. Upon completion of the follow-up assessment, participants received another US $25 incentive.

### Measures

Questions assessing demographics, military history (eg, years of service and rank), and TBI [59] were administered at baseline only. Unless otherwise noted, the following measures were administered at baseline and 30-day follow-up.

#### Pain Intensity

Level of pain was assessed using the widely used 11-point numerical scale of pain intensity [60]. Four versions of the scale asked participants to rate their (1) level of pain right now, (2) usual level of pain in the last week, (3) best level of pain in the last week, and (4) worst level of pain in the last week [61]. All ratings were on a scale of 0 to 10, with 0=no pain and 10=worst pain. Provisional benchmarks for interpreting the clinical significance of change scores on numerical rating scales for pain suggest that reductions of ≥30% appear to reflect at least moderately important improvement.

#### Pain Impact

The Pain Impact Questionnaire (PIQ-6) [62] is a 6-item measure designed to measure level of pain and the impact of pain on work, leisure activities, and well-being. The measure has high internal consistency (Cronbach alpha=.94) and good convergent and discriminant validity. Weighted scores range from 40 to 78, with higher scores reflecting greater pain impact [62].

#### Pain Self-Management Skills

Pain self-management skills were assessed using the MPRCQ2 [56], a 26-item version of the 69-item MPRCQ [57]. Similar to the MPRCQ, the MPRCQ2 assesses readiness to use seven adaptive pain coping skills (exercise, task persistence, relaxation, cognitive control, activity pacing, assertive communication, and using proper body mechanics) and to stop using two maladaptive skills (pain contingent rest and asking for assistance). For adaptive skills, response options range from 1=I am not doing this now, and am not interested in ever doing it, to 7=I have been doing this for a long time (at least 6 months). For maladaptive skills, response options range from 1=I am doing this now and am not interested in ever stopping, to 7=I have not done this for a long time (at least 6 months). Two items assess each subscale, with the exception of cognitive control, which has a total of 10 items assessing five types of cognitive control (types of cognitive control were not examined). Scale scores are computed by taking the mean of the items representing each subscale. Other research has shown that MPRCQ2 subscale scores are highly correlated with subscale scores on the original MPRCQ, associated with readiness to change, and sensitive to change that occurs over the course of traditional treatment for pain. Unfortunately, in this study, two MPRCQ2 items were inadvertently omitted from the measure—one item from the cognitive control subscale and the other from the assertive communication subscale. The score for cognitive control is represented by the mean of the remaining 9 items; the score for assertive communication is represented by the score on the remaining single item.

#### Posttraumatic Stress Disorder

PTSD was measured using the PTSD Checklist—Military Version [63]. This 17-item measure asks how much respondents have been bothered in the past month by each of the 17 *Diagnostic and Statistical Manual of Mental Disorders, Fourth Edition* (DSM-IV) PTSD symptoms related to “stressful military experiences.” Response options range from 1=not at all, to 5=extremely. A total symptom severity score (range=17-85) can be obtained by summing the 17 items. A severity score of 50 has been widely recommended as the cut-off suggestive of PTSD [63]. However, more recent research recommends cut scores as low as 31 [64].

#### Well-Being

Well-being was assessed using the Cantril Self-Anchoring Scale [65], which asks participants to imagine a ladder with steps numbered from 0 to 10, with the top representing the best possible life and the bottom representing the worst possible life, and to indicate where they feel their life falls currently and where it will fall in 5 years.

#### Stage of Change for Pain Self-Management

The stage of change measure for pain self-management was adapted from an algorithm developed in a previous work on pain self-management for patients with interstitial cystitis [66]. Participants were provided with a list of six effective self-management strategies (eg, exercising regularly, controlling negative thoughts about the pain) and asked about their readiness to use at least three of them to manage their pain. Patients who reported that they had no intention of doing so in the next 6 months were classified in the precontemplation stage; those who intended to do so in the next 6 months or next 30 days were classified in the contemplation or preparation stage, respectively. Those who had been meeting the action criteria for less than 6 months were in the action stage, and those who had been meeting criteria for more than 6 months were in maintenance.

#### Stage of Change for Stress Management

Readiness to practice stress management was assessed with a staging algorithm used previously to assess outcomes in a randomized trial of a computerized TTM intervention for stress management [67]. The question defines healthy stress management strategies and asks participants if they effectively practice them. [68]. Response options and scoring rules match those used for pain self-management, described above.

#### Stage of Change for Practicing Healthy Sleep Habits

Readiness to practice healthy sleep habits was assessed using a staging algorithm that provided a list of healthy sleep habits (eg, getting at least 7 hours of sleep a night, maintaining a regular bedtime and wake time, avoiding caffeine, alcohol, nicotine, spicy foods, and heavy meals within 4 hours of bedtime) and asked about the intention to engage in them regularly [69]. Response options and scoring rules match those used for pain self-management, described above.

#### Global Impressions of Change

The Patient Global Impression of Change Scale, administered only at follow-up, is recommended as a core outcome measure in studies of pain [60]. In this study, the scale included seven categorical responses to measure improvement or aggravation of pain. *Since beginning this program, how would you describe the change (if any) in activity limitations, symptoms, emotions, and overall quality of life related to your painful condition?* Response options ranged from 1=No change (or condition has gotten worse), to 7=A great deal better, and a considerable improvement that has made all the difference.

#### Program Usability

At follow-up only, program usability was assessed using the System Usability Scale (SUS) [70,71], a 10-item measure recommended by the Department of Health and Human Service usability.gov resource for assessing the usability of digital content [72]. Respondents were asked to score each of the 10 items (eg, “I felt very confident using the system”) using responses ranging from 1=strongly agree to 5=strongly disagree. Some items were reversed scored. In this study, Cronbach alpha was .89. SUS items were summed and recalibrated to yield a total score ranging from 0 to 100. Across studies, the average SUS score was 68 [73].

#### Program Acceptability

Acceptability was assessed using 10 questions adapted from National Cancer Institute’s Education Materials Review Form [74]. In this study, questions were positive statements regarding participants’ perceptions of the program’s appeal, suitability for veterans, and potential to impact change. Response options ranged from 1=strongly disagree, to 4=strongly agree. Cronbach alpha was .92 in this study. An overall acceptability score for Health eRide was computed as the mean of the 10 items. Additional open-ended questions assessed what participants liked most and liked least about the program, and how the program could be improved.

### Analysis Plan

The first set of analyses assessed pre-post changes in pain, pain impact, pain coping strategies, PTSD, well-being, and measures of stage of change for pain self-management, stress management, and healthy sleep. Pre-post changes on continuous measures were examined using paired samples tests. Stage measures were dichotomized (pre-Action vs Action or Maintenance), and pre-post changes were examined using the McNemar chi-square test with continuity correction. The McNemar test is used for binary dependent variables in a within-subjects design when the same individuals are measured twice. Measures of effect size—Cohen for the continuous outcomes and odds ratios for the binary outcomes—were also computed. The formula for Cohen used here ([M−M]/SD) does not take into account the correlation between the pre- and postmeasures, yielding a more conservative—and accurate [75]—measure of effect size.

Descriptive statistics were computed for program usability and acceptability measures. It was decided at the outset that the criterion for establishing program usability would be a score >68, the average SUS score across studies [73]. The criterion for establishing program acceptability would be an overall mean acceptability score ≥3.

## Results

A total of 69 participants completed an initial study session, which included the CTI and additional study measures. The session lasted an average of 39.3 min (SD 20.0 min). During the next 30 days, 81% (56/69) of the participants completed at least one PAC activity. On average, study participants completed an average of 9.4 PAC activities (SD 11.9). In all, 64% (44/69) opted to receive text messages and 30% (21/69) validated their phone number. During the course of the study, 5 participants texted “Stop” or turned the messages off manually through the Health eRide program.

At baseline, 10% (7/69) screened positive for a TBI. The mean score on the numerical rating scale assessing current pain was 5.8 (SD 2.0). The stage distribution for pain self-management was bimodal: 1% (1/69) of the participants were in the precontemplation stage for pain self-management; 15% (10/69) were in contemplation; 39% (27/69) preparation; 3% (2/69) action; and 42% (29/69) maintenance. Participants selected an average of 2.8 (SD 1.6) MPRCQ2 pain coping strategies to learn more about or work on during their CTI session. They were most likely to select exercise (47%, 32/68), relaxation (47%, 32/68), avoiding pain contingent rest (41%, 28/68), and cognitive control (35%, 24/68).

A total of 44 participants (64%) completed the 30-day follow-up assessment. There were no differences between respondents and nonrespondents on demographics, military history, positive screen for TBI, pain, stage of change for pain self-management, or any other study measures, with the exception of current well-being, assessed using the Cantril Self-Anchoring Scale [65]. Current well-being scores were significantly higher for respondents than for nonrespondents: 6.0 (SD 2.2) versus 4.3 (SD 2.1), respectively, t_67_=2.82, *P*=.006).

### Pre-Post Changes

Results, summarized in Tables 1 and 2, show that pre-post changes in the levels of pain and pain impact, as well as stage of change for pain self-management and stress management reached statistical significance; effect sizes were quite large.

**Table 1:** Pre-post changes on key measures.

Outcomes		Time 1	Time 2	*t*^a^	*P* value	Cohen *d*
		Mean (SD)	Mean (SD)			
**Pain**						
	Pain now	5.8 (2.1)	5.0 (2.0)	3.325	.002	0.395
	Usual pain past week	6.8 (1.6)	5.4 (1.9)	5.117	<.001	0.751
	Best pain past week	4.9 (2.1)	4.0 (2.1)	3.253	.002	0.428
	Worst pain past week	8.3 (1.4)	7.0 (1.7)	4.883	<.001	0.804
Pain impact		65.87 (5.4)	61.5 (7.2)	4.908	<.001	0.673
**Pain coping skills**						
	Exercise	4.5 (1.6)	4.6 (1.5)	−2.273	.03	0.399
	Task persistence	4.5 (1.7)	4.6 (1.6)	−0.271	.79	0.048
	Relaxation	3.8 (1.9)	4.6 (1.7)	−2.835	.007	0.444
	Cognitive control	4.1 (1.3)	5.3 (1.1)	−2.149	.04	0.376
	Pacing	4.9 (1.8)	3.4 (1.4)	−1.445	.16	0.242
	Avoiding pain contingent rest	3.5 (2.1)	3.5 (2.2)	0.222	.83	−0.047
	Avoiding asking for assistance	3.7 (2.0)	4.9 (2.1)	0.446	.66	−0.098
	Assertive communication	4.8 (2.4)	5.5 (2.1)	−0.252	.80	0.041
	Use of proper body mechanics	4.7 (1.8)	4.6 (1.5)	−2.738	.009	0.457
Posttraumatic stress disorder		31.4 (21.5)	27.2 (21.8)	2.008	.05	0.192
**Emotional well-being**						
	Present well-being	6.0 (2.2)	6.3 (1.8)	.966	.34	−0.139
	Future well-being	7.2 (2.1)	7.4 (2.1)	.696	.49	−0.098

^a^Paired samples *t* test, degrees of freedom=43.

**Table 2:** Pre-post changes in percent in action/maintenance.

Target behavior	Time 1, %	Time 2, %	McNemar χ^2a^	*P* value	Odds ratio
A or M stage-pain management	54.5	79.5	6.67	.010	6.500
A or M stage-stress management	50.0	88.6	13.47	<.001	18.000
A or M stage-healthy sleep	25.0	38.6	2.50	.113	4.000

^a^With continuity correction, degrees of freedom=1; N=44.

Using available benchmarks for interpreting the clinical significance of changes in pain intensity ratings (ie, a 30% reduction from pre to post), rates of at least moderately important improvement were 26% (11/43) for current pain, 32% (14/44) for usual pain in the past week, 34% (15/44) for best pain in the past week, and 23% (10/44) for worst pain in the past week. These rates of clinically significant improvement are comparable with those found in other studies of Web-based pain self-management programs (eg, 38% in a study of a Web-based acceptance and commitment therapy intervention [76] and 19% in a study of a 10-week interactive voice response–based CBT intervention [77]; in the latter study, the rate of clinically significant improvement among patients receiving a 10-week in-person CBT intervention was 33% [77]. Changes in pain impact scores correspond to reduction from severe impact to substantial impact and a drop below the national mean for chronic pain patients. The reduction in symptoms of PTSD approached significance (=.05). There were significant increases in four of the nine pain coping skills assessed with the MPRCQ2, which are as follows: exercise, relaxation, cognitive control, and use of proper body mechanics. Pre-post changes in perceptions of current and future well-being and stage of change for practicing healthy sleep habits were not statistically significant.

### Patient Global Impression of Change Scale

When asked to report on their global impressions of change, 41% (18/44) of the respondents reported that they had experienced a slight but noticeable improvement, 11% (5/44) had experienced a definite improvement, and 16% (7/44) said that they had experienced considerable improvement in their condition. Only 32% (14/44) of the participants reported that they had not experienced any noticeable change in their condition or that the change did not make a difference.

**Table 3:** Mean system usability and acceptability scores (N=44).

Usability measure		Mean (SD^a^)
System usability scale score^b^		65.4 (13.3)
Overall acceptability score^c^		3.2 (0.5)
**Ten individual acceptability dimensions**		
	I liked the way the program looked.	3.3 (0.7)
	I enjoyed using the program.	3.2 (0.5)
	Questions were easy to understand.	3.2 (0.7)
	Feedback was easy to understand.	3.3 (0.6)
	Program was interesting.	3.3 (0.6)
	Program was designed for Veterans.	2.8 (0.9)
	Program gave sound advice.	3.2 (0.6)
	Program gave me something new to think about.	3.3 (0.6)
	Program gave me new ideas about managing pain.	3.3 (0.5)
	Program could help me change behavior.	3.0 (0.6)

^a^SD: standard deviation.

^b^Usability criterion: mean system usability scale score ≥68.

^c^On a 4-point scale, acceptability criterion: mean overall acceptability score ≥3.

### Program Usability and Acceptability

Program acceptability and usability ratings are presented in Table 3.

The mean usability score for the Health eRide program was 65.4 (SD 13.3), falling slightly short of the mean score of 68 found across other studies of digital materials [73]. The overall mean acceptability score was 3.2 (SD 0.5), exceeding the criterion score of 3.0 for program acceptability. The lowest mean rating was 2.8 for the statement, “The program was designed for Veterans.”

In response to the question, “What did you like most about the program?” 95% (42/44) described elements they liked and the remainder (5%, 2/44) provided no response. Participants were most likely to comment that they like the information and content, and the ease of use. For example, participants wrote the following:

The program is very easy to use, large print, very intuitive, not a cumbersome program.

It made me consider the things I have done to improve my quality of life with pain...exercise, knowing when to take it easy, sleep, eating better.

All of it really but the steps the program gives is easy to follow in a pace u control at your own pace they [sic] some methods I used and others I am working on.

It not only asked me about my pain and issues, but it also gave me solutions to resolve my issues.

In response to the question, “What did you like least about the program?” 43% (19/44) said “nothing” or described elements they liked. The remainder (57%, 25/44) described elements they did not like. Respondents were most likely to comment on length of the program, confusion on how to answer some of the questions, confusion over the design of the program and the idea that the program did not necessarily provide users with “new” information:

The initial subway hub was confusing and the layout didn’t help.

Some of the questions were a little difficult to answer based on the answer choices.

It seemed to take a lot of questions to get to a conclusion. After I go through everything I am not really sure how to find a particular piece of information that was provided.

Some areas were a little confusing...needed to re-read directions, in order to understand what you were looking for.

In response to the final open-ended question, “How could the program be improved?” 47.7% said, “Nothing” or “Don’t know,” or made a positive comment about the program—for example, “I think it’s fine the way it is.” The remainder (53.3%) offered a recommendation on how the program could be improved. Recommendations included making the program shorter, clarifying instructions and the wording of the questions, and making it more usable on mobile. Respondents also suggested adding audio, videos, or other features. For example, participants wrote the following:

Easier to drill down into the information. [M]ore concise way to get to the root of the problem and give the option for more info. It would also be nice if there was a notebook like feature where you could save parts that interest you for future reference.

I didn’t notice if there was an audio option for the program. This program was not good for mobile use. Might consider a mobile site.

Videos would be a good tool, seeing reactions of real people and how they manage pain the healthy way.

## Discussion

### Principal Findings

This research provides preliminary data on the potential impact, usability, and acceptability of Health eRide, a prototype of a TTM-based mobile intervention for pain self-management among veterans. The data are encouraging. After a single session, at 30 days’ follow-up, participants reported statistically significant reductions in pain intensity and pain impact, and effect sizes were quite large. Benchmarks for interpreting clinical significance of reductions in pain intensity show that around one-fourth to one-third of the participants experienced at least moderately important improvement on the four measures of pain intensity examined. On the Patient Global Impression of Change scale, over one-fourth of the participants reported either definite or considerable improvement in their pain. Patients also showed significant pre-post changes in readiness to engage in pain self-management and stress management and on readiness to use the following four specific pain self-management strategies: exercise, relaxation, cognitive control, and use of proper body mechanics. Three of those strategies were among those that participants most often chose to focus on in their intervention sessions. Reductions in PTSD approached statistical significance (=.05). Whereas the sample’s mean score on SUS fell short of the study’s criterion score for establishing feasibility, the mean score on the acceptability measure exceeded the criterion score for establishing acceptability. Responses to open-ended questions show that some participants particularly appreciated the program’s clarity and ease of use, whereas others found various components (eg, response options, the layout of the subways station) confusing. Additional usability and program refinement will be necessary to ensure ease of use for all participants. Responses to open-ended questions highlight a number of additional opportunities for improvement, including reducing session length (especially the number of measures) and including more videos. In subsequent implementations, additional efforts will be made to further customize the intervention materials to veterans. Reasons for relatively low validation of phone numbers among participants who opted to receive text messages will be explored.

The challenges to recruitment provide lessons for a subsequent randomized trial. First, the lack of follow-through on the screener on Facebook suggests some distrust of an unknown organization asking for contact information. This hypothesis is supported by the Web-based survey company’s success in recruiting, given that respondents had a preexisting relationship with the organization. When recruiting from community sources, it will be critical to have the support and advocacy of an organization that serves veterans to help promote the program from the outset. Second, it may be best to conduct all eligibility screening online, with eligible participants segueing directly to the program log-in page. In some environments, a particularly promising approach may be to integrate the Health eRide program into clinical practice, with provider or clinic endorsement, and the provision of iPad or tablets to support universal Web-based screening and session completion in the waiting room.

Questions may be raised about the role incentives had on veteran’s willingness to participate. In the pilot test, financial incentives (redeemable gift cards) were used to encourage participation. It is not uncommon to incentivize research participants [78], particularly because in this pilot study, they completed additional assessments that would not be included in the real-world implementation of Health eRide. Planned eventual dissemination channels for Health eRide include the Veteran’s Administration and other veteran-service organizations (eg, Tricare); Veteran-centric social networking sites (eg, Rally Point); the app store; and community-based primary care, where there is a new emphasis on nonpharmacological approaches to managing chronic pain [79]. Previous research demonstrates that primary care provider referrals significantly increased adherence to a recommended behavior change intervention, particularly when accompanied by arranging follow-up [80]. Furthermore, in the longer-term clinical trial and in real-world implementations, nonmonetary incentives for participation in this could include the emotional and instrumental support from other participants via social networking; praise for participation provided by the program and by health care providers if the program is delivered in a clinical setting; the sense of mastery provided by progressing through the subway stops to the final destination in the Health eRide program; and, most importantly, the rewards of improved pain and pain self-management.

### Limitations

There are several limitations to this research, including the self-selection bias introduced by the recruitment methods, small sample size, and brief follow-up. Given the bimodal distribution for pain self-management—39% (27/69) in the preparation stage, 42% (29/69) in the maintenance stage), it is safe to say that individuals in the precontemplation and contemplation stages were underrepresented in the study. Concerns are mitigated somewhat by the similarities in age, mean numeric rating of pain, racial and ethnic distribution to the sample recruited by Heapy et al [77]. Another limitation relates to the fact that participation in the 30-day follow-up was predicted by well-being at baseline, with respondents reporting significantly higher well-being than nonrespondents. This may have led to more favorable findings than if all participants had responded.

### Future Work

Future work will include in the completion of the development of Health eRide to address the recommendations from pilot participants and lessons learned, to add other enhancements, and to program additional interactions with input from potential end users and experts in pain management, social networking, and gaming. A randomized trial with longer follow-up will be required to assess the efficacy of the Health eRide program. These preliminary data, however, suggest that Health eRide has the potential to be an important component of an integrated evidence-based approach to pain care among veterans.

## Abbreviations

CBT: cognitive behavioral therapy
CTI: computer tailored intervention
MPRCQ2: Multidimensional Pain Readiness to Change Questionnaire
OEF: Operations Enduring Freedom
OIF: Operations Iraqi Freedom
PAC: Personal Activity Center
PMD: postdeployment multisymptom disorder
PTSD: posttraumatic stress disorder
SMS: short message service
SD: standard deviation
SSI: Survey Sampling International
SUS: System Usability Scale
TBI: traumatic brain injury
TTM: transtheoretical model
VACHS: Veterans Administration Connecticut Healthcare System
VHA: Veterans Health Administration
